# The Effects of Forest Therapy on Immune Function

**DOI:** 10.3390/ijerph18168440

**Published:** 2021-08-10

**Authors:** Youngran Chae, Sunhee Lee, Youngmi Jo, Soyean Kang, Suyoun Park, Hyoyoung Kang

**Affiliations:** 1 College of Nursing, Kangwon National University, Chuncheon 24341, Korea; yrchae@kangwon.ac.kr; 2 Department of Nursing, Yeoju Institute of Technology, Yeoju-si 12653, Korea; 3 Department of Nursing, Kangwon National University Hospital, Chuncheon 24289, Korea; youngs905@hanmail.net; 4 Department of Nursing, Daewon University College, Jecheon 27135, Korea; seizy@daewon.ac.kr; 5 Department of Nursing, Kangwon National University, Chuncheon 24341, Korea; suyoun2419@gmail.com; 6 Department of Nursing, Songho College, Heongseong 25242, Korea; sissy2@songho.ac.kr

**Keywords:** forest therapy, adults, immune function

## Abstract

We conducted a systematic review of the effects of a forest therapy program on adults’ immune function. We used PICO-SD (participants, interventions, comparisons, outcomes, study design) to identify key items. The participants were adults over the age of 18 and the intervention was forest therapy. Our comparisons included studies that comparatively analyzed urban groups or groups that did not participate in forest therapy intervention. Cases without control groups were also included. Immunological outcome measures were included in measuring intervention outcomes. All experimental studies, such as randomized controlled trials (RCTs), non-equivalent control group designs (non-RCTs), and one-group pretest-posttest design were included in the study design. A total of 13 studies were included for comparison. Forest therapy programs were divided into lodging-type and session-type programs. The representative measures for evaluating the effects of immune function were the number of NK cells, the cytotoxic activity of NK cells, and cytotoxic effector molecules. Most studies reported improvement in these measures when comparing values after intervention with values before the forest therapy intervention. Therefore, forest therapy has been found to be effective in improving immune function. More RCT studies on the effects of forest therapy on immune function are necessary.

## 1. Introduction

Forest therapy creates a state of physical relaxation by exposing a participant to a natural environment. It is thought to activate compromised immune function and improve immune function for maintaining and promoting health [1]. Forest therapy makes use of various elements of the forest environment to help individuals cope with stress and to maintain and promote their health [1]. As awareness of forest therapy has increased, so has the number of individuals participating in this therapy [2]. In addition, as stress levels have escalated and public frustration caused by social distancing mandates during the COVID-19 pandemic has increased, public interest in forest therapy has also increased. An increasing number of visitors to forest areas have indicated that COVID-19 was the motivation behind their forest visit [3].

In line with this trend, there has been a steady increase in studies investigating and verifying the effects of forest therapy [4,5,6,7,8,9,10]. Numerous studies have reported that forest therapy can have positive effects on physical and psychological health [4,5,6]. Forest therapy has also been reported to improve depression [7]. Furthermore, forest therapy reportedly reduces sympathetic nervous activity, increases parasympathetic nervous activity, and regulates the balance of autonomic nerves, all of which lead to increased relaxation [8].

Along with its ability to reduce stress, forest therapy has been shown to improve immune function [9,10]. Stress conditions affect immune function [11,12]. In particular, chronic stress suppresses immune responses and promotes pathological immune responses, including inflammatory responses [13,14,15]. Thus, if forest therapy can reduce stress, it will simultaneously enhance immune function. Moreover, some studies [16,17] have reported that environmental factors have a greater impact on immune function than genetic factors. This indicates that environmental characteristics, such as those provided by forest therapy, can have a positive effect on immune function.

However, although studies of forest therapy have used various outcome measures related to immune function [18,19,20,21,22], such as NK cells, T cells, B cells, perforin, granulysin, granzymes, and interleukin-6, their results show inconsistently. Therefore, it is necessary to identify the effective measures among the various outcome measures of immune function and to analyze which outcomes of the immune function measurements show changes due to the intervention of forest therapy.

In addition, several researchers have presented a systematic review of studies on the health effects of forest therapy [4,5,14,23], but few have presented a systematic review on the effect of forest therapy on immune function specifically. Thus we present a systematic review of studies on the effects of forest therapy on the immune function of adults, investigating the characteristics of forest therapy programs and analyzing their effects on immune function measures.

## 2. Materials and Methods

### 2.1. Inclusion and Exclusion Criteria for Selection of Existing Studies

This study was conducted according to the guidelines of the Preferred Reporting Items for Systematic Review and Meta-Analyses (PRISMA) [24]. The publication year of the article was not limited. Key items of the criteria for selecting existing studies for this study, were composed according to PICO-SD (participants, intervention, comparisons, outcomes, and study design): (1) The participants were adults aged over 18 years; (2) The intervention included forest therapy; (3) That the studies compared forest therapy groups, with groups that did not participate in forest therapy intervention or urban groups, and cases without control groups were also included; (4) Outcomes of the intervention included immunological outcome measures, and (5) In terms of study design, all experimental studies, such as randomized controlled trials (RCTs), non-equivalent control group designs (Non-RCTs), and one-group pretest-posttest design were included. Studies without experimental designs, such as survey research and qualitative research, were excluded from the analysis.

### 2.2. Searching for and Selecting Existing Studies

We included studies that could be retrieved in each database in our analysis following a search conducted in the two weeks between 1 July and 14 July 2020. For domestic databases, RISS, KISS, DBPia, and NDSL were used, and for international databases, PubMed, Cochrane library, PsychoInFO, EMBASE, EBSCO, Web of Science, CINAHL, and Scopus were used. The references of the searched articles were used to perform a manual search in addition to the electronic search for collection. The search keywords were (“shinrin-yoku” OR “forest bathing” OR “nature therapy” OR “forest therapy”) AND (“Immunity” OR “Natural killer cell” OR “NK cell” OR “immune”). For domestic keywords, Korean was used with the same meaning as the English keywords. Only studies published in English or Korean were included.

Each database was searched following a discussion between the two researchers, and one of the researchers deleted duplicate articles using a document management program. The title and abstract were then reviewed according to the inclusion and exclusion criteria, to screen the articles. Once an article passed the initial screening stage, its full text was checked, and only those that met each of the selection criteria were selected. In the case of any disagreement during this process, the two researchers had a discussion and reached a mutual consensus regarding the final selection of each article.

### 2.3. Risk of Bias Assessment of Individual Studies

To assess the selected articles’ risk of bias, we used the Revised Cochrane Risk-of-Bias tool (RoB 2) [25] for RCTs, while the Risk-of-Bias Assessment tool for Non-randomized Study (RoBANS) [26] tool was used for Non-RCTs.

The risk-of-bias assessment tool for RCTs is composed of the following five domains: randomization process, deviations from intended interventions, missing outcome data, measurement of the outcome, and selection of the reported result. The risk of bias assessment for these domains was performed using three categories: “low risk”, “some concerns”, and “high risk”.

The RoBANS is composed of the following six items: selection of participants, confounding variables, measurement of intervention (exposure), blinding for outcome assessment, incomplete outcome data, and selective outcome reporting. The risk was assessed using “low risk”, “high risk”, and “uncertain risk” categories. “uncertain risk” means that the study is judged to raise some concerns in at least one domain, but not to be at high risk of bias for any domain. In this study, two researchers independently performed a quality evaluation. When there was a disagreement between the two researchers, included a third-party researcher in their discussion to help bring them to an agreeable conclusion.

### 2.4. Data Extraction

Items for data extraction included study information (author, publication year), participants (total number of participants, age, and diagnosis), study design and intervention program characteristics, measurement tools, main outcome variables, and ethical considerations.

## 3. Results

### 3.1. Study Selection

**Figure 1:**
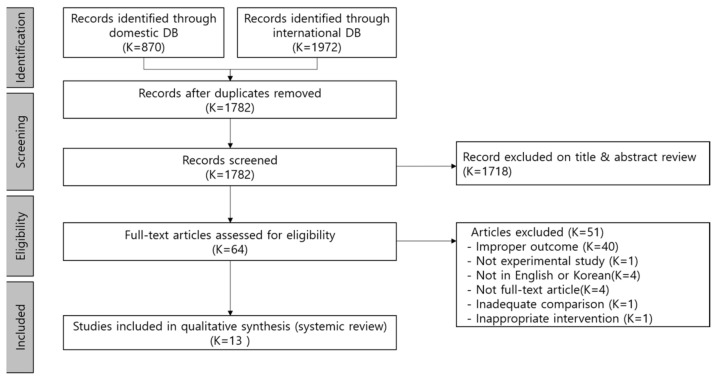
PRISMA flow chart.

We retrieved 870 articles from domestic databases and 1972 articles from international databases for our analysis. Once duplicate articles were removed, 1782 articles remained. The titles and abstracts of each of these articles were reviewed, and 1718 articles did not comply with the inclusion and exclusion criteria. Finally, 64 articles remained after the screening process. The full text of each of these articles was reviewed, and the following 50 articles were excluded in total: 40 articles that contained no report on immunological outcomes, one article with non-experimental study, four articles that were not published in English or Korean, four articles whose original version could not be retrieved, one article with inadequate comparison, and one article on indirect forest therapy (aromatherapy). Thus, only 13 peer-reviewed articles were selected for further analysis (Figure 1).

### 3.2. Characteristics of the Existing Studies

In terms of the selected existing studies’ publication year, there were no reports published before 2006, while there were three articles published between 2007 and 2010 [18,19,20], four articles between 2011 and 2015 [21,22,27,28], and six articles from 2016 to present [29,30,31,32,33,34]. With regard to participants, six of the studies used healthy adults as participants [18,19,20,21,27,34], while five had participants who were adults with health problems [22,28,29,30,31], and two did not report as to their participants’ health status [32,33]. In terms of countries/regions where the study was conducted, four studies were conducted in Korea [27,28,30,32], China [21,29,31,34], and Japan [18,19,20,22], respectively, and one study was conducted in Taiwan [33]. As for the study design, three studies were RCTs [21,29,31], five studies used non-equivalent control group pre-posttest design [20,27,30,32,34], and five studies used a one-group pretest-posttest design [18,19,22,28,33]. In terms of sample size, there were seven studies with a sample size of 20 or less [18,19,21,28,29,32,33], four studies with a sample size between 21 and 50 [20,22,27,31], and two studies with a sample size of 51 or larger [30,34]. With regard to ethical considerations, apart from one study with no description of Institutional Review Board (IRB) approval status [32], the other 12 studies were all approved by the IRB (Table 1).

### 3.3. Characteristics of the Forest Therapy Program

We found that “forest bathing” was the most commonly used term in five articles, followed by “forest therapy”, used in four articles, while “visiting forest”, “green space”, “forest environment”, and “forest walking” were each used in one article, respectively.

The forest therapy programs were classified into lodging-type and session-type programs. For lodging-type programs, seven studies lasted for 2–3 days [18,19,20,21,27,30,34], three studies that lasted 4–5 days [29,31,33], and one study [28] that lasted for 14 days. For session-type programs, two studies were conducted for 12 weeks [22,32], one of which held a session weekly [22] while the other held sessions three times weekly [32]. There were eight studies in which the intervention only included walking in the forest [18,19,20,21,29,31,32,33], while four studies used meditation, horticultural therapy, yoga meditation, support group therapy, music, cognitive-behavioral therapy, and relaxation therapy as interventions, alongside walking in the forest [22,27,28,30]. One study [34] provided no specific description of the intervention but simply mentioned forest exposure.

There were eight studies in which the participants were healthy adults and five studies in which the participants were adults with health problems. The healthy adult participants included college students [19,21,34], middle-aged women or men [18,33,34], nurses [19], and workers in the healthcare and counseling service industries [27]. As for the participants who were adults with health problems, two studies included cancer patients [22,28], one included patients with chronic obstructive pulmonary disease [29], one included chronic pain patients [30], and one study’s participants were patients with congestive heart failure [31] (Table 2).

### 3.4. Effects of Forest Therapy on Immune Function

With regard to immune function measures, the number of NK cells or NK cell activity was the most frequent measure used in the studies we reviewed. The number of NK cells was reported in nine studies [18,19,20,21,28,29,32,33,34]; a significant increase in NK cell count was reported in six studies [18,19,20,28,32,34], and no significant change in the number of NK cells was reported in three studies [21,29,33].

NK cell activity was measured in eight studies [18,19,20,22,27,30,33,34], and a significant change was reported in seven studies. The study that reported no significant change in the activity of NK cells [27] applied a combination of interventions, including walking, meditation, counseling, cognitive-behavioral therapy, and music therapy for three days and two nights in the forest, for workers in the healthcare and counseling service industries. In a study with female nurses as participants, which used a walking intervention of two hours in the morning and afternoon, respectively, for three days and two nights [19], a significant increase in both the number and activity of NK cells was reported, for up to 7 days after returning from the forest. In addition, in a study with healthy male adults as participants which used a walking intervention of two hours each morning and afternoon, respectively, for three days and two nights [20], a significant increase in the number and activity of NK cells was reported for not only up to 7 days after the intervention, but also 30 days after returning from the forest.

T cells were measured in four studies [18,19,20,21] and showed no significant changes in three of these studies [18,20,21]. In a study with healthy male university students as participants, where the intervention included 90 min of walking in the morning and afternoon, respectively, for two days [21], B cells, T-helper cells, suppressor cells, and NK cells were measured; a significant change was observed only in B cells. In a study in which patients with chronic obstructive pulmonary disease participated, that included 90 min of walking intervention in the morning and afternoon, respectively, for four days [29], no significant changes were seen in the Natural Killer T(NKT)-like cells or CD8+ T cells.

Six studies [18,19,20,28,29,34] measured granulysin, perforin, and granzymes A and B, which are cytotoxic effector molecules. In a study with male college students as participants [34], there was no significant change in granulysin, and in a study with chronic obstructive pulmonary disease patients as participants [29], there was no significant change in granzyme B, while significant changes in these measures were reported in four other studies.

Three studies [21,29,31] measured the levels of proinflammatory cytokines. In a study that used a walking intervention for healthy male university students [21], IL-6 and TNF-α were measured, and significant changes were reported in both. In a study that used 90 min of walking intervention for patients with chronic obstructive pulmonary disease, in the morning and afternoon, respectively, for four days in the forest [29], IL-6, IL-8, IFN-γ, IL-1β, and TNF-α were measured; significant changes were reported in each of these outcome measures apart from TNF-α. In a study that used a walking intervention for chronic heart failure patients for four days in the forest [31], there was a significant change seen in IL-6 levels, but no significant change was reported in TNF-α levels (Table 3).

### 3.5. Risk of Bias Assessment

**Figure 2:**
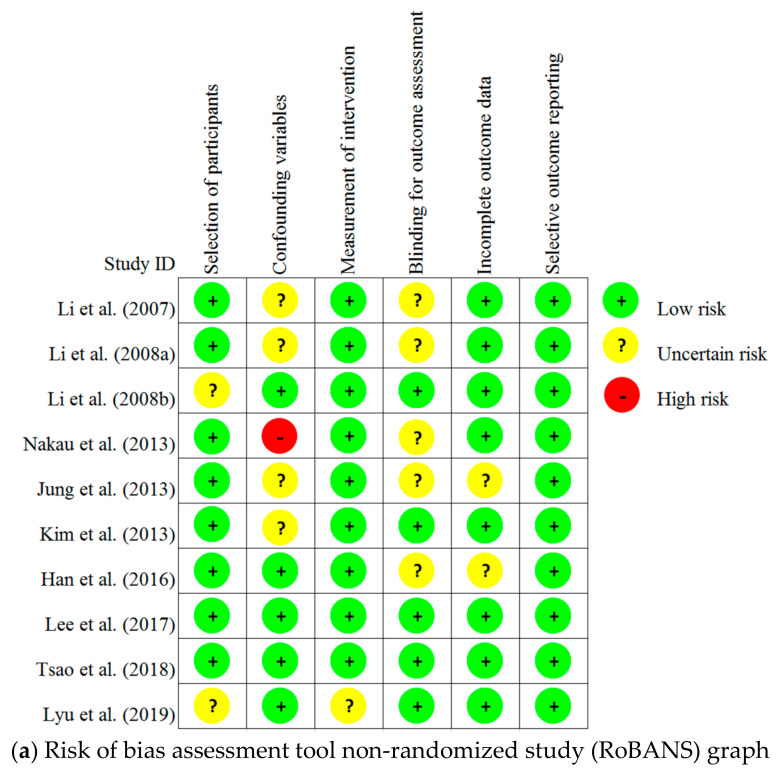

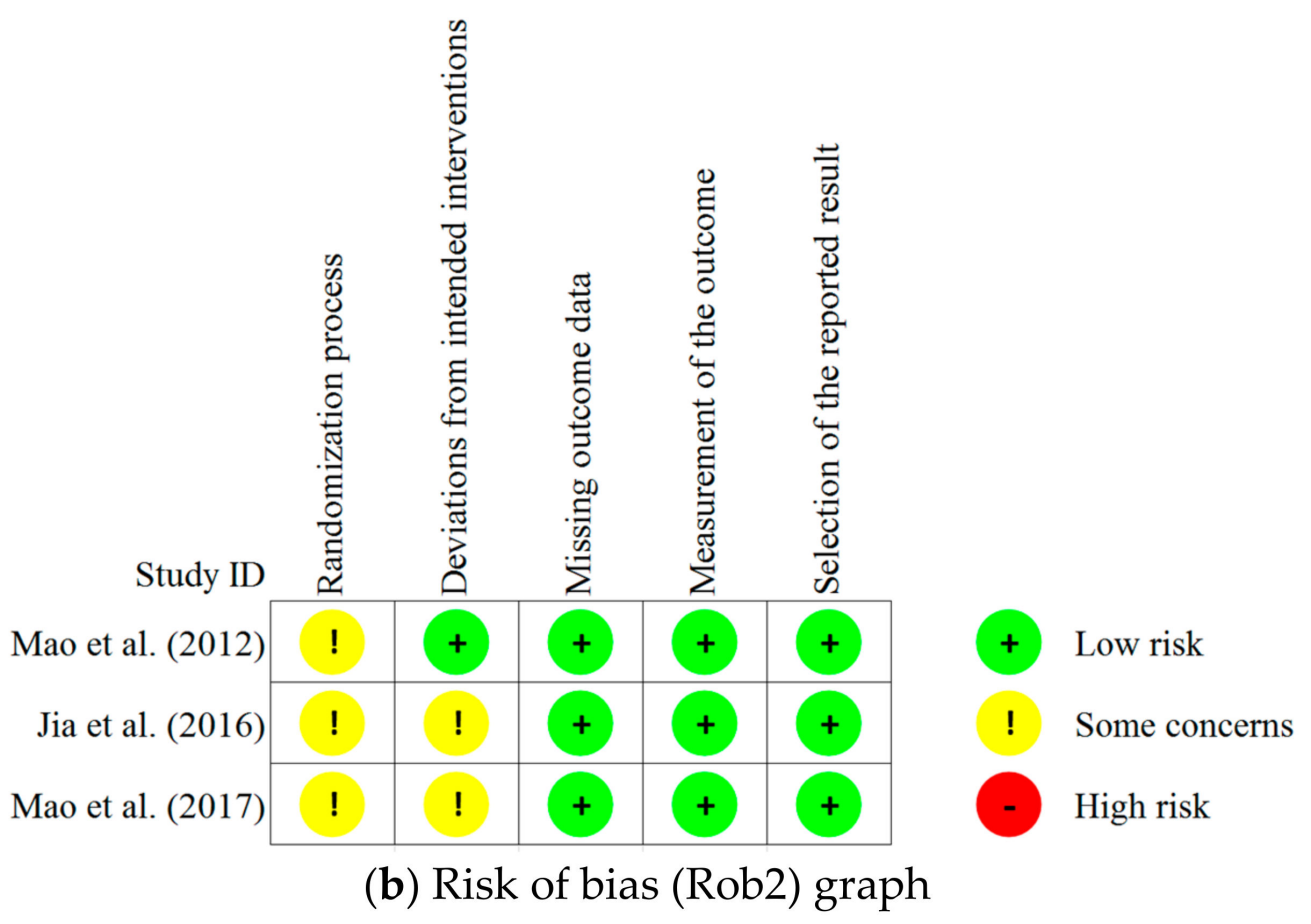
Risk of bias in included studies.

The results of the risk of bias assessment we conducted on the 13 articles reviewed in this study are presented in Figure 2.

With regard to the three RCTs, none of the three articles included a detailed description of the randomization process and thus were assessed as having “some concerns” in the category of Randomization Process. In terms of Deviations from Intended Interventions, no information on dropouts was presented and the dropouts were not included in the analysis of two of the RCTs; thus, these two studies were assessed as having “some concerns,” while the other RCT was assessed as “low risk.” In terms of Missing Outcome Data, Measurement of the Outcome, and Selective Outcome Reporting, all three studies were assessed as “low risk.”

Of the 10 non-RCT studies, two were assessed as of “uncertain risk” because the recruitment criteria of the patient group and control group were not consistent in terms of Selection of Participants, and the rest were assessed as “low risk.” In terms of Confounding Variables, one study was assessed as “high risk”, because there was no clear description of the management of NK cells, and multiple items of subjective quality assessment were related to the intervention and determined as the factors affecting the outcomes. Further, four studies were assessed as being of “uncertain risk” because they gave no clear description of the control of variables nor any clear information on the exclusion of the time elapsed, considering the intervention. The remaining studies were assessed as “low risk.” In terms of Measurement of Intervention (Exposure), one study was assessed as being of “uncertain risk” because it did not give a sufficiently detailed description of the intervention (exposure) method other than naming the place of intervention, while the other nine studies were assessed as “low risk.”

In terms of the Blinding for Outcome Assessment, five studies were assessed as being of “uncertain risk” because it was unclear whether the blinding status would affect outcome measurements, while the other studies were assessed as “low risk.” In terms of Incomplete Outcome Data, two studies were assessed as being of “uncertain risk”, because they did not have sufficient information on missing data, and the other studies were assessed as “low risk.” In terms of Selective Outcome Reporting, all 10 studies included all expected outcomes, and they were assessed as “low risk”.

## 4. Discussion

This systematic review of studies on the effects of forest therapy on immune function aims to identify the characteristics of forest therapy programs and to analyze the effects of forest therapy on immune function outcome measures.

Since 2006, all studies have investigated the effects of forest therapy on immune function. An increasing number of studies have been conducted within the last five years, indicating a recent surge of interest in the effects of forest therapy on immune function. However, among the studies published so far, one group of pre-post test design studies accounted for a high proportion (38.5%), and the sample size of each of the studies we reviewed was small. This indicates that a low level of evidence is provided by these studies. In order to produce more systematic and scientific results, a more stringently controlled study design will be required in future research.

Examining the details of the forest programs reveals that 61.5% of the studies only used a forest walking intervention and no other interventions. In fact, the majority of studies used a walking intervention, meaning that the main component of forest therapy was walking. This finding is consistent with the results of a previous forest therapy study [35], in which walking accounted for a major part of the forest therapy program’s composition.

In terms of the intervention period, programs were classified into lodging-type (lasting anywhere from three days and two nights to 14 days) and session-type programs. No program operated as a short-term one-off type, as reported by Chae et al. [6], indicating that an intervention period of a minimum of two nights and three days is required for the improvement of immune function and maintenance of improved outcomes.

To date, there have been few standardized forest therapy programs and insufficient individual forest therapy studies, posing difficulties in clearly identifying the most effective intervention method [36]. Only 13 articles were included in this systematic review and the studies presented were highly heterogeneous and thus unsuitable for meta-analysis. Consequently, we were only able to conduct a systematic review of these existing studies. If more individual studies are accumulated in the future, we will perform a meta-analysis according to intervention type and duration, which will enable a more objective evaluation of the effects of forest therapy on immune function.

We considered the number of NK cells, NK activity, and cytotoxic effector molecules as measures to evaluate forest therapy’s effects on immune function. In 12 of the reviewed studies, the number of NK cells and/or NK activity was reported. NK cells are capable of attacking and killing virus-infected cells or tumor cells and play an important role in the human endocrine and immune systems [37]. It is thought that NK cells were often measured in these studies because, in the forest environment, the activity of NK cells is enhanced by an increase in the number of NK cells and cytotoxic effector molecules, leading to enhanced immune function [18,38]. In general, NK cells are highly important lymphocytes [39] that serve as a first-line defense against virus-infected cells. They rapidly proliferate in the stress of transient acute exercise but are vulnerable to chronic stress. Among the lymphocyte subtypes (T cells, B cells, and NK cells), NK cells are known to be most responsive to exercise intensity [40].

In this study, six of the nine studies that reported the number of NK cells reported a significant increase in the number following forest therapy intervention, and out of the eight studies that reported the activity of NK cells, seven reported a significant increase in NK cell activity following forest therapy intervention. In particular, two studies with healthy adults [19,20] revealed that the number and activity of NK cells continued to increase significantly, for up to seven days or longer after returning to the urban environment, while a study [20] with healthy male adults showed a significant increase in NK cell activity up to 30 days after the intervention. In other words, the results indicate that forest therapy can have a long-term effect on the number and activity of NK cells in the human body. The study [28] on an urban woman with breast cancer who had received anti-cancer treatment suggested the potential of forest therapy as adjuvant anti-cancer therapy after standard treatments. However, since both healthy adults and adults with health problems were included in the studies that showed a significant change and the studies that did not show a significant change, and because the type of forest therapy was different in different studies, our investigation of the difference in the effects according to the characteristics of participants and the types of forest therapy programs was limited.

Of the seven studies that analyzed cytotoxic effector molecules such as perforin, the majority reported significant changes in the outcomes. This could be because NK cells secrete perforin and granzymes [41,42], and granulysin [43,44] through the granule exocytosis pathway, which leads to the destruction of tumor cells or virus-infected cells. Activation of NK cells via the release of perforin and granzymes is important for inducing natural cytotoxicity [45]. Studies in perforin-deficient mice indicated that NK cell-mediated cytotoxicity is greatly impaired in such mice [46]. The NK cells of mice with a deficiency in the granzyme B cluster, induce apoptosis in target cells more slowly than wild-type NK cells [47].

A number of studies [1,7,8,38,48,49] reported that volatile substances (phytoncides) extracted from trees have a positive effect on immune function, supporting the idea that a forest’s environmental factors play an instrumental role in improving immune function. An in vitro study indicated that certain volatile tree chemicals, called phytoncides, increase the activation of NK cells and intracellular anti-cancer molecules [50]. This assertion is supported by several studies in animals [51,52] and humans [53], suggesting that fragrances from trees can reverse stress-induced immunosuppression, and normalize immune function and neuroendocrine hormone levels.

Stress increases sympathetic nervous system activity and hypothalamus-pituitary-adrenal system activity to increase cortisol secretion. However, phytoncides have positive effects on stress reduction, cortisol level reduction, blood pressure reduction, immune system enhancement, autonomic nervous system, and chronic fatigue, without side effects [54]. It has also been reported that inhalation of phytoncide through breathing during forest bathing, or smell, can enhance the cytotoxic activity of NK cells [55]. In addition, NK cells are increased by the decreased production of stress hormones and the increased production of anticancer proteins caused by phytoncides [49].

Several studies have analyzed cytokines such as interferon-γ, interleukin-1β, and tumor necrosis factor α or lymphocyte subtypes, but it was difficult to determine outcomes from their results due to their small number. However, in the case of T cells, previous research suggests that careful consideration is required while selecting T cells for verification of forest therapy effects in the future, as three out of four studies included in this review reported that there were no significant changes in T cells.

One limitation of this study is that it analyzed only articles written in English and Korean, and included non-RCT studies as well as RCT studies that have a high level of evidence. In addition, since only a small number of studies were included, another limitation was found in presenting the effect size according to the characteristics of the participants or the program used. However, this systematic review is significant in that it suggests that there is evidence to support the theory that forest therapy can have a positive effect on immune function.

## 5. Conclusions

The results of this review recommend the use of the number or activity of NK cells for evaluating the effects of forest therapy on immune function, and cytotoxic effector molecules are also thought to serve as effective outcome measures. Forest therapy programs, including walking in the forest, may contribute to the improvement of immune function, and forest therapy is expected to be utilized for the enhancement of immune function in the future.

More RCT studies on the effects of forest therapy on immune function are necessary, to strengthen the body of evidence to support the use of forest therapy for improving immune function.

## Figures and Tables

**Table 1:** General characteristics of included studies (*n* = 13).

Characteristic	Categories	N (%)
Publication year	≤2006	0 (0)
	2007–2010	3 (23.1)
	2011–2015	4 (30.8)
	≥2016	6 (46.2)
Participants	Healthy adults	6 (46.2)
	Adults with health problems	5 (38.5)
	Not reported	2 (15.4)
Country/Regions	China	4 (30.8)
	Korea	4 (30.8)
	Japan	4 (30.8)
	Taiwan	1 (7.7)
Study Design	Randomized control group	3 (23.1)
	Nonequivalent control group pre-posttest design	5 (38.5)
	One group pre-post test design	5 (38.5)
Sample size	≤20	7 (53.8)
	21–50	4 (30.8)
	≥51	2 (15.4)
Statement of ethical consideration	Yes	12 (92.3)
	No	1 (7.7)

**Table 2:** Summary of studies included in the systematic review (k = 13).

	Authors (Years)	Study Design	Participants (n)	Intervention	Control	Outcome (Measurements)
1	Li et al. (2007) [18]	One group pre-posttest design	Healthy male, aged 37~55 years (12) Mean age: 43.1 ± 6.1	A three-day/two-night trip First day: walked for two hours in the afternoon in a forest field Second day: walked for two hours each in the morning and afternoon in two different forest field On day 3: finished the trip	None	· NK cells **↑, NK activity **↑, · T cells · granulysin **↑, perforin **↑, granzymes A **↑/B **↑ · WBC, (Lymphocytes *↑, Monocytes*↑, Granulocytes **↓) · T-A *↓, D *↓, A-H, F, C, V **↑(POMS) · the hours of sleep
2	Li et al. (2008) [19]	One group pre-posttest design	Healthy female nurses, aged 25~43 years (13) Mean age: 28.8 ± 4.6	A three-day/two-night trip First day: walked for two hours in the afternoon in a forest field Second day: walked for two hours each in the morning and afternoon in two different forest field On day 3: finished the trip	None	· NK cells **↑, NK activity **↑ · T cells *↓ · granulysin **↑, perforin **↑, granzymes A **↑/B **↑ · Adrenalin **↓, noradrenaline (Urine) **↓ · Estradiol, progesterone · T-A, D **↓, A-H, F, C, V (POMS)
3	Li et al. (2008) [20]	None-equivalent control group pre-posttest design	Healthy male, aged 35~56 years (E:12, C:11) Mean age: 45.1 ± 6.7	A three-day/two-night forest bathing program First day: walked for two hours in the afternoon in a forest field Second day: walked for two hours each in the morning and afternoon in two different forest field On day 3: finished the trip	A three-day/two-night city trip First day: walked for two hours in the afternoon in an old-style district in the city Second day: walked for two hours around baseball Dome in the morning and 2 h around/in airport in the afternoon On day 3: finished the trip	· NK cells *↑, NK activity *↑ · T cells · granulysin **↑, perforin **↑, and granzymes A **↑/B **↑ · WBC · Adrenaline (Urine) *↓
4	Mao et al. (2012) [21]	RCT	Healthy male university students (E:10, C:10) Mean age: 20.79 ± 0.54	two-night trip walked for 1.5 h each in the morning and afternoon in a forest field	walked for 1.5 h each in the morning and afternoon in a city site	· NK cells · T cell, B cell *↑, T-helper cells. suppressor cells, natural killer cells · IL-6*↓, TNF-α *↓ · T-SOD, MDA **↓ · ET-1 **↓, Platelet activation · Cortisol *↓, Testosterone · T-A *↓, D *↓, A-H *↓, F *↓, C, V *↑(POMS)
5	Nakau et al. (2013) [22]	One group pre-posttest design	Breast cancer or lung cancer (22) Mean age: 58.1 ± 10.8	Walking in the forest, Horticultural therapy, yoga meditation, and support group therapy, and sessions were conducted once a week for 12 weeks	None	· NK activity **↑ · Well-being of functional and spiritual (FACIT-Sp): GP, GS, GE, GF *↑, Sp *↑ · Quality of life (SF-36): PF *↑, RP *↑, BP, GH *↑, VT **↑, SF, RE *↑, MH *↑ · Fatigue (CFS) **↓ · T-A *↓, D, A-H, F, C **↓, V (POMS) · STAI: State anxiety **↓, Trait anxiety *↓
6	Jung et al. (2015) [27]	None-equivalent control group pre-posttest design	Workers in the healthcare and counseling service industries (E:19, C:20) Mean age E: 29.42 ± 8.92, C:36.45 ± 12.23	3 days, 2 nights, Walking and meditation in the forest, and exposure to a psychological program using music and cognitive-behavioral therapy.	frequently use the environment without participating in the program	· NK activity · Cortisol *↑ · HRV · MBI-GS **↓, WRSI *↓, REQ **↑
7	Kim et al. (2015) [28]	One group pre-posttest design	Stage 3 Breast cancer patient, aged 25~60 years (11) Mean age: 56 ± 5.12 (11)	Stay in the forest for 14 days, Forest therapy program (Walk, forest life)	None	· NK cells **↑ · Perforin *↑, Granzyme B *↑
8	Jia et al. (2016) [29]	RCT	elderly patients with chronic obstructive pulmonary disease (E: 10, C: 8) Mean age E: 70.1 C: 70	4 days, walking in the forest (total 3 h walk/day)	4 days, walking in the city (total 3 h walk/day)	· NK cells, NKT-like cell, CD8+ T cells · Perforin **↓, Granzyme B · IL-6 *↓, IL-8 **↓, interleukin-1β *↓, TNF-α interferon-γ *↓, CRP *↓ · Cortisol *↓, Epinephrine *↓ · RARC/CCL-18 **↓, TIMP-1 *↓, SP-D *↓ · T-A **↓, D *↓, A-H *↓, F, C, V (POMS)
9	Han et al. (2016) [30]	None-equivalent control group pre-posttest design	Full-time employees with chronic widespread pain for more than three months, aged 25~49 years (E: 33, C: 28) Mean age E: 41.6 ± 6.5, C: 37.5 ± 8.4	2 days forest therapy program Walking and therapeutic activities in the forest activities (music therapy, psycho-education: coping with pain and stress, bodily exercises, mindfulness-based meditation)	The control group was instructed not to conduct either heavy loads of domestic or occupational work during the enrollment in this study.	· NK activity **↑ · Heart rate variability (HRV) **↑ · Self-reported pain **↓ (VAS) · Depression **↓ (BDI) · Health-related quality of life **↑ (EQ-VAS)
10	Mao et al. (2017) [31]	RCT	Elderly patients with chronic heart failure, Aged from 65 to 80 years (E: 23, C: 10) Mean age 73.86 ± 5.85 years old	4 days, Walking in the forest (total 3 h walk/day)	4 days, Walking in the city (total 3 h walk/day)	· IL-6 *↓, TNF-α, CRP · BNP **↓, N-terminal pro BNP · ET-1 **↓, Renin, AGT, Ang II, AT1, AT2 **↑ · T-SOD *↑, MDA *↓ · T-A *↓, D **↓, A-H **↓, F, C **↓, V (POMS)
11	Lee et al. (2017) [32]	None-equivalent control group pre-posttest design	Women in their 50 s (E: 9, C: 9) Mean age E: 53.9 ± 2.69, C: 55.5 ± 1.84	Forest walking exercise for 12 weeks (3 times/week, 110 min/day)	Ground walking exercise for 12 weeks (3 times/week, 110 min/day)	· NK cells *↑ · Melatonin *↑
12	Tsao et al. (2018) [33]	One group pre-post test design	Middle-aged subjects (11) Mean age: 60.4	A five-day/four-night trip (maintain dietary control and walking exercise) First day: walked for 1.5 h in the afternoon in a forest field Next three days: walked for 1.5 h each in the morning and afternoon in two different forest field Fifth day: finished the trip	None	· NK cells, NK activity **↑
13	Lyu et al. (2019) [34]	None-equivalent control group pre-posttest design	Male College Students (E: 45, C: 15) Mean age E: 20.9 ± 0.24, C: 21.3 ± 0.45	Bamboo forest site exposure for three days	City site exposure for three days	· NK cells *↑. NK activity *↑, · granulysin, perforin *↑, granzymes A *↑/B *↑, · corticosterone *↓ · SBP *↓, DBP, HR, SpO2 · T-A *↓, D *↓, A-H *↓, F *↓, C *↓, V *↑ (POMS)

*: *p* < 0.05; **: *p* < 0.01; ↑: indicators rise; ↓: indicators decline; E = Experimental group; C = Control group; POMS = Profile of mood states; T-A = Tension-anxiety; D = Depression: A-H = Anger-hostility,; F = fatigue; C = Confusion; V = Vigor; WBC = hite blood cell; IL-6 = Interleukin-6; TNF-α = Tumor necrosis factors α; T-SOD = Total superoxide dismutase; MDA = Activity and malondialdehyde; ET-1 = Endothelin-1; FACIT-Sp = Functional assessment of chronic illness therapy-spiritual; GP = Physical well-being; GS = Social/family well-being; GE = Emotional well-being; GF = Functional well-being; Sp = Spiritual well-being; CFS = Cancer fatigue scale; SF-36 = 36-Item short-form health survey; PF = Physical functioning; RP = Role-physica; BP = Bodily pain; GH = General health perception; VT = Vitality; SF = social functioning; RE = Role emotional; MH = Mental health; STAI = State-trait anxiety inventory; HRV = Heart rate variability; MBI-GS = Maslach Burnout Inventory-General Survey; WRSI = Worker’s Stress Response Inventory; REQ = Recovery Experience Questionnaire; CRP = C-peptide protein; RARC/CCL-18= Pulmonary and activation-regulated chemokine, TIMP-1= Tissue inhibitor of metalloproteinase-1; SP-D = Surfactant protein D; VAS: Visual analog scale; BDI = Beck Depression Inventory; EQ-VAS= EuroQol Visual Analog Scale; BNP = Brain natriureti; AGT = Angiotensinogen; Ang II = Angiotensin II; AT1 = Angiotensin II type 1 receptor; AT2 = Angiotensin II type 2 receptor; SBP = Systolic blood pressure; DBP = Diastolic blood pressure; HR = Heart rate.

**Table 3:** Summary of the effects of forest therapy on immune function.

Outcome		Article Number	
		Significant	Not Significant
Number of NK cells		18, 19, 20, 28, 32, 34	21, 29, 33
NK cells cytotoxic activity		18, 19, 20, 22, 30, 33, 34	27
T cells		19	18, 20, 21
B cells		21	
T suppressor cells			21
T-helper cells			21
Natural killer T(NKT) like cells			29
Cytotoxic T cells			29
Cytotoxic effector molecules	granulysin	18, 19, 20	34
	perforin	18, 19, 20, 28, 29, 34	
	granzymes A	18, 19, 20, 34	
	granzymes B	18, 19, 20, 28. 34	29
Proinflammatory cytokines	interleukin-6 (IL-6)	21, 29, 31	
	interleukin-8 (IL-8)	29	
	interferon-γ (IFN-γ)	29	
	interleukin-1β (IL-1β)	29	
	tumor necrosis factor α (TNF-α)	21	29, 31

## Data Availability

No new data were created or analyzed in this study. Data sharing is not applicable to this article.
